# Temporal-Spatial Analysis of the Warming Effect of Different Cultivated Land Urbanization of Metropolitan Area in China

**DOI:** 10.1038/s41598-020-59593-0

**Published:** 2020-02-17

**Authors:** Jiayang Li, Chunxiao Zhang, Xinqi Zheng, Youmin Chen

**Affiliations:** 1 School of Information Engineering, China University of Geosciences, Beijing, 100083 China; 2 Polytechnic Center for Territory Spatial Big-Data, MNR of China, Beijing, 100036 China; 3 The College of Environment and Planning of Henan University, Kaifeng, 475004 China

**Keywords:** Climate and Earth system modelling, Environmental impact

## Abstract

The regional warming effect is different when different cultivated land types are converted into urban construction land, while its temporal and spatial changes are unclear. We studied the temporal and spatial changes in the warming effect when dry land was converted to urban land (DL2UBL), and irrigated land to urban land (IL2UBL) in Yangtze River Delta (CSJ), Beijing-Tianjin- Hebei (JJJ) and Chengdu-Chongqing (CY) metropolitan areas from 2000 to 2015. The average warming effect of the three metropolitan areas was more intensive in DL2UBL than in IL2UBL in winter, and opposite occurred in summer. The diurnal warming changes between them were small during the day, but obvious at night, which corresponds to the diurnal change of the latent heat of evaporation. Due to the difference in the spatial distribution of humidity, to the north of 34°N, the warming effect of DL2UBL was stronger than that of IL2UBL, and to the south of 34°N, it was stronger for IL2UBL, while from west to east, the warming trend of DL2UBL and IL2UBL keep pace and decline slightly. The influence in planetary boundary layer was also analyzed. We hope that our findings provide scientific support for future metropolitan land use decisions associated with tradeoffs.

## Introduction

Human activities are most frequent in cities and with the development of urbanization, by 2030, it is estimated that 82% of the global population will live in cities^1^. Urban expansion is one of the most significant land-use and land-cover changes (LUCC), affecting many aspects of climate^2–4^. For example, Freitag, *et al*.^5^ found that urbanization in complex terrain modified convection and rainfall, and the exchange of heat, moisture, momentum and albedo^6–8^. Moreover, urban warming effects due to urban sprawl have widely concerned in scientific community over decades^9–11^. It is not only because of the anthropogenic heat emission from the vehicles, power factories, and other sources changing air greenhouse gas concentration, but also, due to the LUCC changes to surface features that alter biophysical processes directly^12,13^.

Rapid urbanization in recent decades, especially in developing countries like China, has resulted in large reductions in city cropland area^14,15^. Although croplands and cities result from human transformation of nature, the change in the area of these two underlying surfaces is also an important contributor to climate change^16^. Zhang, *et al*.^17^ found a significant increase in the urban heat island (UHI) spatiotemporal influence by comparing the non-urban scenario of being replaced by irrigated land and the real-life scenario in the Yangtze River Delta. Wang, *et al*.^18^ also found the temperature of newly developed urban areas was higher than before the LUCC in metropolitan Beijing-Tianjin-Hebei, which had previously been mainly the cultivated land^19^. Given such extensive study, they usually regarded cultivated land as one land-use class^14,20–22^ and there is a little quantitative study on the specific cropland land-use conversion to urban. However, clear cognition into the temporal and spatial changes of the difference concerning different cropland is significant in China. On the one hand, because of marked climatic differences due to the vast land area, cultivated land is usually divided into two classifications: dry land (no irrigation water source and facilities, mainly cultivated vegetables, DL) and irrigated land (cultivation of rice, lotus root and other aquatic crops with water supply and irrigation facilities, IL). On the other hand, there are different properties between the two kinds of cultivated land^23^, which should result in the different warming effect caused by the conversion of two cultivated land into urban and built-up land (UBL).

The warming effect that results from cultivated land urbanization is a process accompanied by a complex changes in biochemical and biophysical processes, however, most studies on these changes have focused on surface temperature or land surface temperature as the primary metric^24,25^. Nevertheless, because moisture as one of the main factors influencing the warming effect, some recent study focused on the analysis including temperature and moisture^26,27^. Pielke ^28^ used the concept of heat content, including the latent heat of evaporation and moisture, to quantify global warming in the ocean, and an effective temperature was introduced from the heat content concept to assess the warming effect of surface air change. Ideally this could be compared with air temperature and it was appropriate for measuring global warming^24^. However, studies that advance an understanding of the warming influence of different cropland urbanization patterns from a heat content perspective are scarce.

In regard of the different properties of cropland and its different effect on warming, by investigating the humidity and temperature, this study studied the warming effect of conversion from different cultivated land into UBL in the Yangtze River Delta area (named ChangSanJiao in Chinese, CSJ), Beijing-Tianjin-Hebei agglomeration (the abbreviation from the province name is Jing-Jin-Ji in Chinese, JJJ) and Chengdu-Chongqing urban groups (the abbreviation from the province name is Cheng-Yu in Chinese, CY), which are multi-cities combination of spatial units that were jointly planned. Our approach used fine land use classification data as the unique variable and coupled with a WRF-UCM model to quantitatively explore the warming effect caused by different cropland urbanization types in the three important Chinese metropolitan areas in 2000 and 2015. We expected that this spatiotemporal quantification results will further clarify the source and extent of regional warming and support the metropolitan area development planning in the future.

## Results

### WRF Model validation

**Figure 1:**
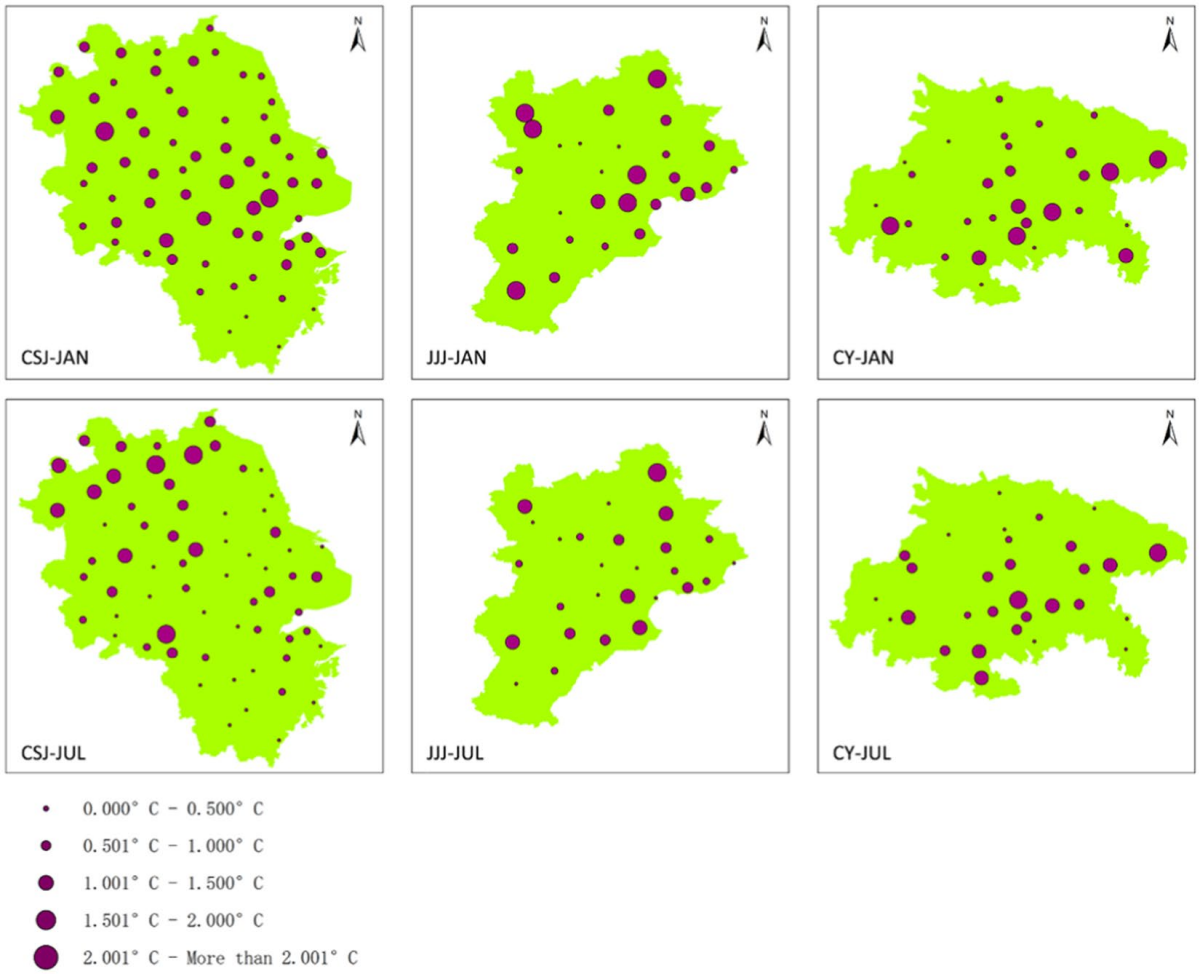
The differences between simulation results at 2-m temperature and meteorology station air temperature in three metropolitan area. The purple dots are the station locations.

To validate the effect of the model simulation, we compared with the CTL (described in Method section) temperature results and the meteorological station temperature data that mentioned in Observation data. Figure 1 shows the location of each meteorological site in the study area, and also monthly average 2-m high temperature difference between the simulating outcome at meteorological sites and station records. The simulation differences of three metropolitans are about 1.0 °C (Fig. 1). More than 70% of sites in JJJ and CY are within 1.0 °C, and the proportion of CSJ sites was more than 80%.

We not only compared the data at each site, but also compared the daily average 2-m temperatures over January and July. The first day of a month as spin-up, was not used in the analysis, rather the data from 2nd to 31st of January and July. Relevant error statistics of Root Mean Square Errors (RMSE), Mean Relative Error (MRE) and Correlation Coefficients (CC) are given in Table 1. RMSE and MRE, in particular, well describe the magnitude of the error between the simulated and observed values. The RMSE of the three experiments was 0.787 °C–1.769 °C and the MRE 0.615 °C–1.572 °C, which is similar to some relevant conclusions^29,30^. CC can express the tightness and consistency of relationship between the daily observations and the simulated values, and for the three domains are about 90%, which shows that the model simulated values reflect the actual change very well^31^.

**Table 1:** Validation indicators of simulated data and observation data. RMSE: Root Mean Square Errors, MRE: Mean Relative Error, CC: Correlation Coefficient.

	RMSE		MRE		CC	
	JAN	JUL	JAN	JUL	JAN	JUL
CSJ	1.443	0.787	1.244	0.615	96.3%	92.3%
JJJ	1.769	0.812	1.572	0.715	88.7%	87.8%
CY	1.212	1.442	0.903	1.190	91.2%	96.3%

### Temperature changes for different urbanized cultivated land

In China, dry cropland (DL) and irrigated land (IL) are the two main land-use types being converted to urban and built-up land (UBL)^32^. The grids of conversion from dry land to urban and built-up land (DL2UBL) and irrigated land to urban and built-up land (IL2UBL) were extracted in the three metropolitan area respectively (Study Area). The 2-m high air temperature (T2) changes are shown in Table 2 (at the 95% confidence level). Conversely, we obtained the temperature change of the cropland region, where there was no land-use change (DL was still DL, IL was still IL).

**Table 2:** The average variation of T2 rise without ambient change after urbanization from different cultivated land types, and the maximum and minimum temperature increases in January and July, respectively are shown.

		JAN			JUL		
		Mean	Max	Min	Mean	Max	Min
CSJ	DL2UBL	0.148 (0.018)	0.07 (0.022)	0.109 (0.008)	0.445 (0.059)	0.137 (0.053)	0.002 (0.007)
	IL2UBL	0.152 (0.039)	0.05 (0.029)	0.003 (0.003)	0.436 (0.115)	0.141 (0.024)	0.249 (0.007)
JJJ	DL2UBL	0.193 (0.013)	0.18 (0.007)	0.001 (0.001)	0.362 (0.047)	0.207 (0.049)	0.008 (0.004)
	IL2UBL	0.25 (0.04)	0.389 (0.008)	0.357 (0.002)	0.419 (0.004)	0.974 (0.338)	0.854 (0.002)
CY	DL2UBL	0.177 (0.022)	0.165 (0.035)	0.024 (−0.001)	0.499 (0.033)	1.131 (0.622)	0.082 (0.001)
	IL2UBL	0.163 (0.029)	0.153 (0.046)	0.178 (0.003)	0.43 (0.057)	1.069 (0.41)	0.595 (0.005)

Ambient temperature change was also shown in parentheses, which was the T2 change from areas without LUCC (°C).

Urbanization of all three regions has resulted in an increase in temperature over the past 15 years, and the increase in summer was higher than in winter. Extreme temperature also rose, and in summer were significantly higher than in winter. Overall, the extreme value growth of IL2UBL was larger than that of DL2UBL, especially in the JJJ region. The mean T2 from IL2UBL in JJJ grew the most in winter (0.25 °C), and in summer was from DL2UBL in CY (0.449 °C) (Table 2). For different cultivated land change area in winter, the area where average T2 of DL2UBL and IL2UBL grids rose the most was JJJ (0.183 °C), followed by CY(0.143 °C), and the smallest increase was CSJ (0.117 °C). To accurately characterize the overall warming effect of the three urban metropolitans, the average temperatures were weighted averages of the urbanized grids in the two types of cultivated land. In summer, the largest increase in T2 was in CY (0.412 °C), followed by JJJ (0.336 °C), and the smallest in CSJ (0.321 °C). In terms of changes in the three urban metropolitan areas, the biggest difference between DL2UBL and IL2UBL was in JJJ.

**Figure 2:**
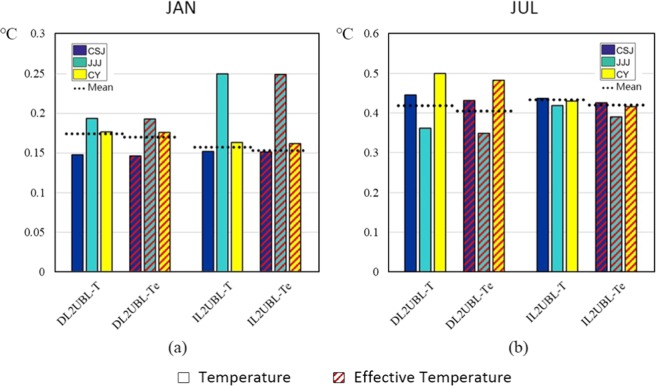
The 2-m temperature and effective temperature difference of cropland urbanized in DL2UBL and IL2UBL in January (**a**) and in July (**b**).

Compared to the air temperature (T_2_), the effective temperature (Te) is a more comprehensive measure of heat storage changes to the earth system^28^ (detailed description in Data Resource and Processing). T2 and Te are compared in January (Fig. 2a) and in July (Fig. 2b). The increase of Te is a little lower than T2 in winter (0.001 °C) and higher in summer (0.013 °C), because of the decline in latent heat of evaporation and specific humidity reducing after urbanization. The correlation coefficient of temperature increase between them is more than 99%, which shows that the trend towards surface changes impact the change of T2 and Te similarly. Overall, considering the latent heat of evaporation and the specific air humidity, the effect of temperature rise caused by the conversion of cultivated land into urban construction land is lower than when considering the temperature alone.

Since the effective temperature combines more factors and can better represent the trend of temperature change with urbanization from different cultivated lands, it was mainly used to analyze the warming effect. From Fig. 2, whether it is DL2UBL or IL2UBL, the warming effect is obvious. Compared to the warming effect in winter, the Te increase of DL2UBL (0.17 °C) is larger than IL2UBL (0.157 °C), however, in summer it is lower by 0.017 °C than the increase of IL2UBL (0.423 °C). Therefore, the warming range of different cultivated land urbanization has a significant seasonal effect. For the different metropolitan areas, there is also a difference between seasons. In winter, the warmest is JJJ, especially in the IL2UBL, followed by CY and then CSJ. In the summer, the warmest is CY, followed by CSJ and then JJJ.

### Effect of cultivated land change on diurnal temperature

**Figure 3:**
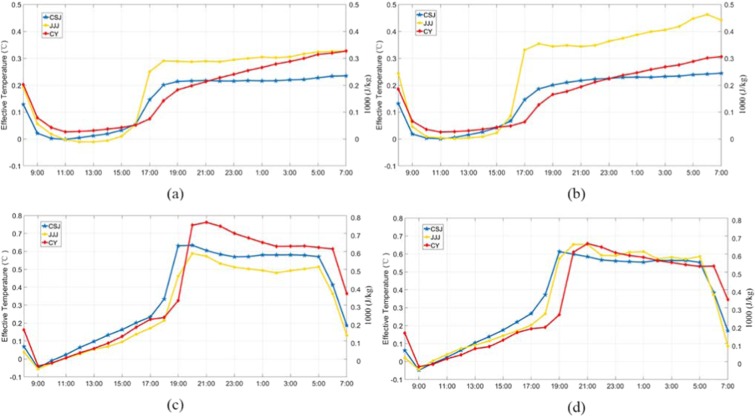
The diurnal effective temperature change of three metropolitan areas of UL2UBL in January (**a**) and of IL2UBL in January (**b**), and the change of DL2UBL in July (**c**) and of IL2UBL in July (**d**).

Although the average temperature can reflect the increasing temperature trend of a region, the diurnal temperature change and range reflects more information for climatic research^33^. There is a correlation between the change of diurnal temperature change and the period of sunlight exposure whether in summer or in winter (Fig. 3). In winter, before receiving solar radiation and making the surface radiation budget balance (about 9:00–15:00), the temperature difference between cultivated land and UBL remains low (about 0 °C). After 15:00–17:00, the warming rate begins to increase gradually and reaches a stable level (0.2 °C–0.35 °C) until midnight. It varied in summer, when it reached its lowest point about 09:00, which is earlier than that in winter, then increased gradually until reaching its highest at noon. After that, the warming increase is maintained above 0.5 °C until 5:00 the next day. However, whether it’s winter or summer, the rapid growth of Te difference occurs after 15:00, until 7:00 of the next day, which will be longer in the summer until 5:00 of the next day.

The increased changes of IL2UBL and DL2UBL, were similar to seasonal changes but different for each metropolitan area. In winter, the Te of cultivated land to UBL in JJJ rose more than others. Average daily temperature of DL2UBL increased by 0.192 °C and IL2UBL increased by 0.248 °C, respectively. CSJ and CY, in southern China, had smaller average of changes of 0.15 °C, as shown in Fig. 2. The change of IL2UBL and DL2UBL impacting on Te were not obvious before 15:00, but after that they grew sharply, especially in JJJ (Fig. 3a,b). In summer, the average growth of IL2UBL and DL2UBL Te was higher, especially in CY, where the Te increased the most and exceeded DL2UBL by 0.75 °C, however, the increase in CSJ was 0.634 °C, and in JJJ was 0.589 °C. For the IL2UBL, the growth rates were similar of three regions in daytime (up to 0.641 °C), and the difference appeared with increasing in the night time.

### Spatial distribution of cropland urbanization warming

**Figure 4:**
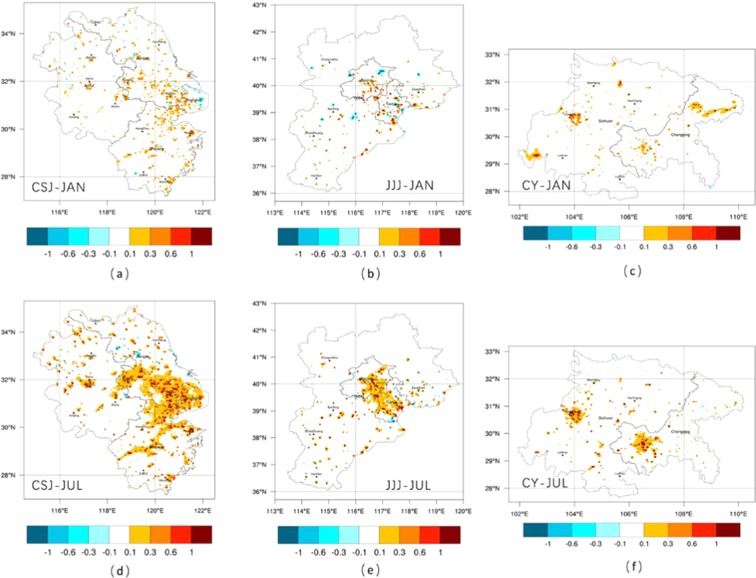
The simulation result of 2-m air temperature difference between the cultivated lands urbanized from 2000–2015. The differences in January in CSJ (**a**), JJJ (**b**) and CY (**c**), as well as it in July in CSJ (**d**), JJJ (**e**) and CY (**f**).

The three metropolitan areas all underwent obvious urbanization processes, and the built-up area of cities expanded, which lead to local and regional temperature increases. Urbanized area, especially the area transformed from cropland to built-up land around existing cities, warmed significantly (Fig. 4). Spatially, the obvious areas where temperature rose were regional centers, such as Shanghai, south of Jiangsu province, north and middle of Zhejiang province in CSJ, the area around Beijing and Tianjin in JJJ, and the area around Chengdu and Chongqing in CY. There was also a strong correlation between the conversion of cultivated land into UBL and temperature increase^19^, which caused 0.017 °C, 0.003 °C and 0.012 °C increase in January (Fig. 4a–c) of each metropolitan, and 0.046 °C, 0.021 °C and 0.017 °C increases in July (Fig. 4d–f), respectively.

**Figure 5:**
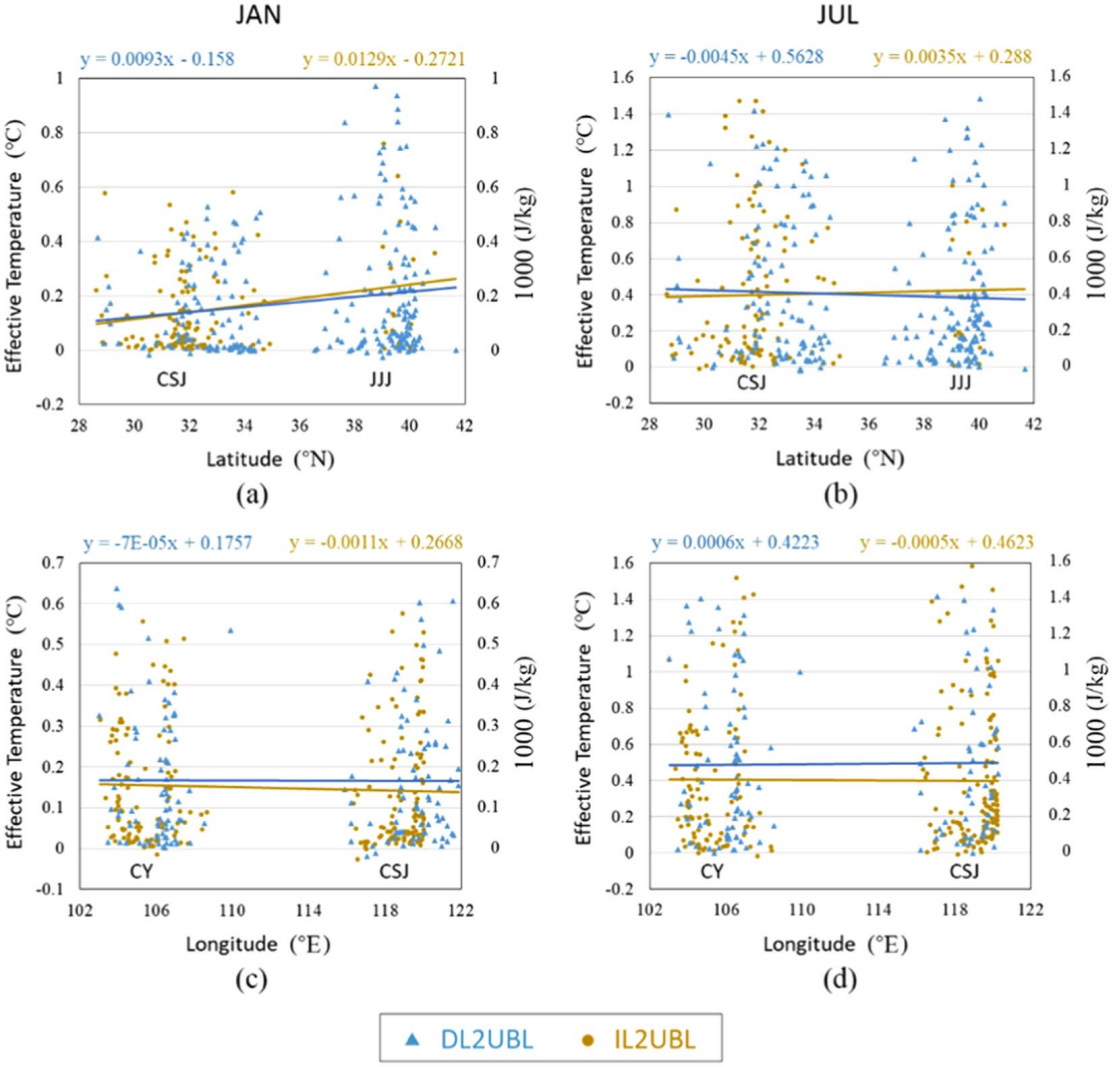
The effective temperature warming trend with latitude and longitude change for different cultivated land converted to UBL. Each point represents a statistical unit of DL2UBL or IL2UBL. The Te change of temperature rise with latitude in January and July are in (**a**,**b**) respectively, and with longitude in January and July in (**c**,**d**), respectively.

To further understand spatial changes in the warming effect by different cultivated land urbanization, we explored a larger scale, considering that CSJ and JJJ are similar in longitude, and CSJ and CY are similar in latitude (as shown in Fig. 5). Combined with the change of humidity and surface latent heat, the Te change of each grid with latitude and longitude was analyzed with linear regression to show change trends. As shown in Fig. 5(a,b), the variation of DL2UBL and IL2UBL grids for overlapping longitude areas CSJ and JJJ were extracted. After the urbanization of the two kinds of cultivated land, the warming range change with increasing latitude shows different characteristics. The Te rise shows an increasing trend with latitude in January no matter whether DL2UBL or IL2UBL, which means urbanized cultivated land in winter is hotter in north, than in south of China in winter. With each degree of increasing latitude, the DL2UBL area would increase 0.013 °C, while it only rose 0.009 °C for the IL2UBL area. In July (summer), the Te trend differs with increasing latitude, with a 0.004 °C increasing of IL2UBL but decrease 0.005 °C in DL2UBL.

With increasing longitude, as shown in Fig. 5(c,d), the variation of DL2UBL and IL2UBL grids in overlapping latitude CSJ and CY areas were extracted, and their warming trends with longitude were fitted. Te rising with increasing longitude shows a consistent and small change. In winter, whether it is IL2UBL or DL2UBL, the heating value decreases by 0.001 °C in IL2UBL and is lower in DL2UBL. Similarly, in July, change in the Te heating value of Te is very small, an order of magnitude lower than the change with increasing latitude.

### Vertical temperature change of cropland urbanization

**Figure 6:**
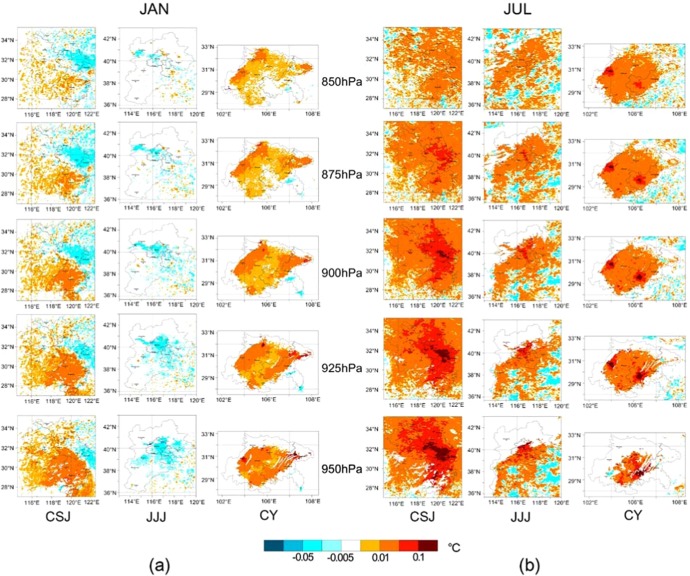
The potential temperature difference of cropland urbanization in different pressure layers in three agglomerations in January (**a**) and July (**b**).

Cultivated land urbanization not only changes the local near-surface meteorological field and thermal balance, but it also affects the entire agglomeration boundary layer. To understand the impact of different cultivated land urbanization on the vertical temperature variation, the spatially vertical potential temperature difference distribution at different pressure levels were shown in Fig. 6. From 950 hpa to 850 hPa with 25 hPa interval of each level, they are all within the planetary boundary layer (PBL) from near to far from the surface. It was obvious that the farther from the ground, the smaller the potential difference. This showed that the vertical influence of LULC change gradually weakened with the distance from the surface. Compared with the near-surface temperature difference (see Fig. 4), the temperature difference was smaller at the levels farther from the surface. The difference was almost within 0.01 °C in winter and within 0.05 °C in summer, and only exceeded 0.1 °C in the surrounding areas of big cities, such as Shanghai, Nanjing in CSJ, Beijing, Tianjin in JJJ and Chengdu, Chongqing in CY. At the top of the PBL, the potential difference across the region was approaching the same.

**Figure 7:**
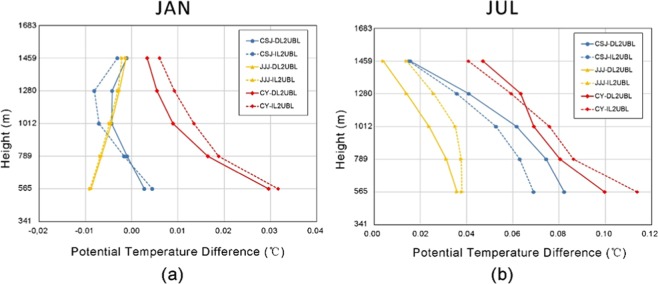
The vertical profiles of potential temperature difference change of two kinds of cultivated land urbanization in three agglomerations in January (**a**) and July (**b**).

The averaged potential temperature difference of DL2UBL and IL2UBL were shown in Fig. 7. The difference of two kinds of cultivated land urbanization were similar in the PBL. In January, the potential temperature difference in CY increased, and increased firstly then decreased in CSJ. In JJJ, the difference was reduced, but the variation decreased as height increased. In July, the potential temperature difference increased in all three agglomerations, but the increase reduced with height increasing. The largest increase in potential temperature difference was in CY, followed by CSJ, and then JJJ area, which was similar to the change in January. As Fig. 7 shown, there was a tendency that the potential temperature difference gradually decreased to 0 °C with height increasing. Comparing to the difference of potential temperature of DL2UBL and IL2UBL, the two kinds of difference in impact were same as those near the surface but smaller than near the surface (see Fig. 2).

## Discussion

Possible reasons for different results using observed data and simulations, are as follows. Firstly, meteorological station data only records the climatic conditions at one point, while simulations cover 3 km × 3 km, which is equivalent to the mean of the region, so the spatial scales of the two comparison data do not match completely. Secondly, it is possible that the initial meteorological and boundary condition are different. Although it has been proven it has little influence in long time simulations^34^, our objective was to compare the differences in simulation results of two sets of LULC. As all other settings are the same, except the surface data (LUCC), the existing errors can be considered as a systematic error of the model and can be offset when analyzing the difference^35^. Compared with earlier studies, the temperature increase of urbanized cultivated land is significantly higher in summer than in winter, which is consistent with some previous research results in China^36,37^. The average increase T2 is 0.162 °C in winter and 0.429 °C in summer in our simulation, which is similar to that documented by Han, *et al*.^38^. In their study, the temperature difference between urbanized, and agricultural land in China was analyzed using meteorological station data from 1960 to 2006. They found a average temperature increase of 0.157 k in May–Sep and 0.347 k in Oct–Apr, which was a little lower than our results, probably because China’s urbanization process was slow from 1960–1990. In the terms of effective temperature Te, Fall, *et al*.^25^ comparing the difference between Te the T2, verified the difference was larger in summer than in winter, as we found. Comparison with previous research shows that our results are credible.

**Figure 8:**
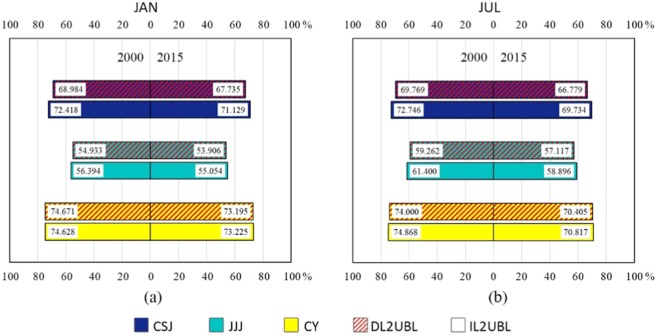
The 2-m relative humidity of three metropolitan areas in 2000–2015 in January (**a**) and in July (**b**).

The trend of temperature change of cultivated land conversion in winter is lower than in summer. This may be related to climate conditions in China, where it is dry in winter and wet in summer (see Study Area section). In winter, total average Te increase for DL2UBL is higher than IL2UBL, while it is opposite in summer, which should be because in winter, IL humidity is higher than DL, causing it to be warmer due to the retention of warmth effect. When they were both converted to UBL, IL2UBL temperature rise is lower than that of DL2UBL. The 2-m relative humidity (see Fig. 8) also shows a lower temperature rise in IL2UBL is larger than DL2UBL. The temperature rose the most in winter and the least in summer in JJJ, which was probably because DL is the main type of cultivated land in the JJJ area where the moisture is much higher in summer than winter. As Shen, *et al*.^39^ pointed out in their study that there was a greater increase in temperature in dry places because of low evaporation. While in summer, the ability of dry land to control the temperature rise is not as strong as irrigated land due to sufficient soil moisture content, higher specific heat capacity results in lower temperature rise, and higher evaporation process removes a large amount of heat and therefore decreases the temperature^19,40^, which causes a higher Te rise in IL2UBL than in DL2UBL. But the Te increase in the CY where IL is the main cultivated land in IL2UBL is lower than in DL2UBL, especially in July (Fig. 2). The main reason could be that high atmospheric moisture emits significantly long-wave radiation (water vapor greenhouse effect), resulting in an enhanced the net heat wave in the atmosphere^26^, and a lesser increase in conversion to UBL. This phenomenon not only occurs in China, but is also pronounced in India^41^.

**Figure 9:**
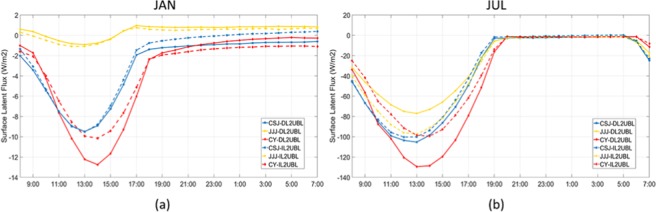
The surface latent flux difference in 2000–2015 of three metropolitan areas in January (**a**) and in July (**b**).

Our diurnal fluctuation are consistent with the previous results^35,42^. Wang, *et al*.^35^ found surface urban agglomerations in China were warmer from18:00 to 6:00 of the next day, the time period of weak solar radiation, and rose less in the day time, which is similar to our result. As shown in Fig. 3, the temperature increases slightly at noon in winter, but occurred all the day time in summer. Surface energy accumulates during the day time, and is released at night. In UBL, due to low humidity, and large diurnal temperature fluctuations, the temperature rise of cultivated land transformed to UBL increased significantly at night^43^. In terms of the surface heat flux, the direct factor affecting diurnal Te change is the latent flux, which also was indicated the most obvious change of heat flux^35^. The diurnal changes in surface latent flux (see Fig. 9), decrease with solar radiation change, which causes a partial reduction in evaporation over the urban land^44^. At noon, when the solar short radiation is the strongest, and the earth’s surface absorbs the most energy, latent heat flux change is at its greatest. Latent flux change between DL2UBL and IL2UBL, decreases more in the JJJ IL2UBL area, but is greater in the CSJ and CY DL2UBL areas. This reduced flux partially enhances the sensible heat of surface air and strengthens the warming effect, which is why in JJJ, the Te rise in IL2UBL is higher than DL2UBL, and is the opposite in CSJ and CY (see Fig. 2).

In JJJ, although the DL2UBL total average temperature rises above that of IL2UBL in winter and it is opposite in summer, the Te of IL2UBL rises more than DL2UBL in JJJ whether it is in winter or in summer. Because JJJ is in northern China, the humidity is lower than in the south, such as in CSJ and CY (see Fig. 6). The higher humidity of IL will reduce heat by evaporation, making the temperature lower than DL and raising the temperature more after being urbanized. Conversely, the moister air will not only increase the moist static energy, absorbing total heat from the wet surface^45^, which makes the DL Te higher. Because CY is in southwestern China, humidity there is significantly higher than JJJ, resulting in the IL2UBL Te increasing less than DL2UBL. In CSJ, the Te change trend is consistent to JJJ in winter and CY in summer (see Fig. 2), and its change is the smallest among them. This is probably because abundant water vapor and high specific heat capacity in the eastern coastal area of China in winter and the smaller change in temperature, meaning that growth rate is lower than the inland CY metropolitan area. However, in summer, as the southeast monsoon strengthens, there is more precipitation in the interior^46^, which reduces the difference. The warming DL2UBL and IL2UBL trend with latitude intersects about 34°N whether in winter or summer (see Fig. 5), where the Qinling Mountain and Huaihe River is located, and where the climate boundary of humid and sub-humid regions between North and South China^47^ occurs, dryer in the north and wetter in the south^48^. South of 34°N, the DL2UBL Te increase is higher than IL2UBL, and to the north of the line, the DL2UBL Te increase is lower than that of IL2UBL, which also verifies the results of our studies. In the terms of the Te change with longitude, the rising trend of DL2UBL and IL2UBL keeps pace, and slightly decreases, probably because the CSJ is next to the sea with large specific heat capacity.

DL2UBL and IL2UBL increased the near-surface temperature as discussed before and they changed the temperature at the atmospheric boundary layer. The closer the surface, the greater the impact (see Fig. 6), which also pointed out by the previous study^49,50^. Ning, *et al*.^51^ also found the PBL height would increase where urban land sprawl in the south of China and the increase of potential temperature decreased the PBL stability but developed the turbulence, especially in the lower part of the PBL. The two kinds of cultivated land urbanization influence in vertical profiles was statistical analysis (see Fig. 7). The influence of the DL2UBL and IL2UBL on PBL was smaller than on the near-surface, this because with the rise of the height, the air mass flew fast, making the heat of the grid homogeneous with surrounding air mass^52^. The impact of land use would be very small at the top of the PBL, so the temperature rise will be gradually decrease to 0 °C as the height increased.

## Conclusion

The warming effect of two kinds of main cultivated land, dry land and irrigated land, urbanized in three important metropolitan areas of China (CSJ, JJJ and CY) were identified by WRF/UCM modeling coupled with fine-scale spatial resolution of 2000 and 2015 LULC data. Overall, in three metropolitan areas, the warming effects of DL2UBL was more intensive than that of IL2UBL in winter and the opposite happened in summer. This is closely related to the effect of water vapor in the air on temperature. For diurnal change of warming effect, the change trend is similar in that the increase is lower in the day time and higher at night, which corresponds to the diurnal change of the latent heat of evaporation, and the contrasting difference for each metropolitan area was greatest at night. There are regular spatial warming effect the changes in latitude and longitude in China. Due to the difference in the spatial distribution of humidity, to the north of 34°N, the warming effect of DL2UBL is stronger than that of IL2UBL, and to the south of 34°N, this phenomenon is reversed, while from west to east, the warming trends of DL2UBL and IL2UBL keeps pace and slightly decrease. Vertically statistical analysis the impact of DL2UBL and IL2UBL on PBL, the two kinds of impact are similar to those occurred near the surface but smaller than near the surface. With the increase of height, the potential temperature difference caused by the change tends to 0 °C.

The warming effect of DL2UBL and IL2UBL in three metropolitan areas of China were carefully studied, however, there are two aspects in need of improvement in future research. Firstly, to analyze the warming effects from different cultivated land conversion to UBL, the main emphasis was on biophysical processes, and biochemical processes were not addressed. Therefore, the impacts of anthropogenic activities and crop growth on carbon cycle and nitrogen cycles were not taken into account. Secondly, there are still some limitations in the simulation results of the three regions, for example, for the control of variables, the simulation parameters are set the same, but the surface characteristics and climate environment of each regions were not identical. A precise simulation of the climate impacts of various LUCCs in each metropolitan area is equally significant for future study to improve regional projections of future climate change and support the urban planning and sustainable development.

## Methods

### Study area

**Figure 10:**
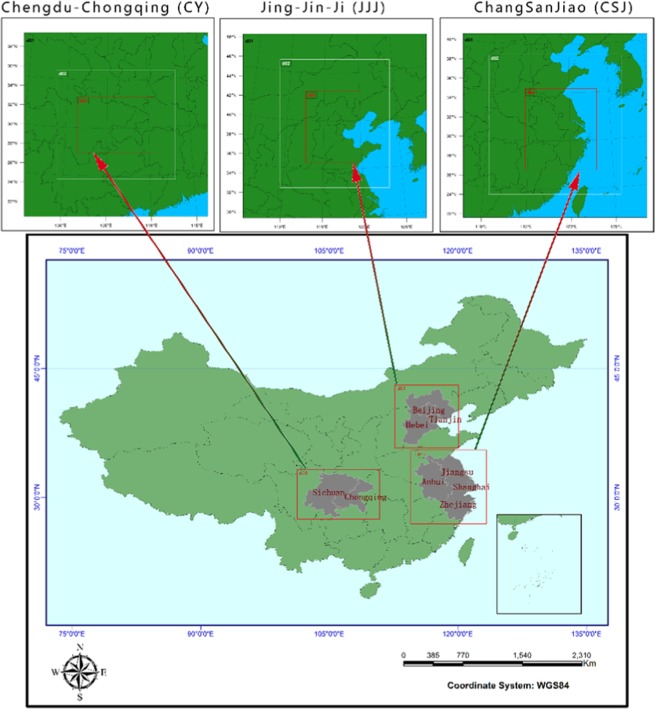
The location of the three metropolitan areas in China, and the three domains of each metropolitan area used in research simulations. The abbreviations of Beijing, Tianjin, Hebei are Jing, Jin, Ji, respectively, in China, so the Beijing-Tianjin-Hebei area we called JJJ. The Yangtze River Delta area is often called “ChangSanJiao” in the Chinese phonetic alphabet, so it is called CSJ. Chongqing is abbreviated as Yu, so the Chengdu-Chongqing region is called CY.

CSJ, one of the most mature metropolitan area in China, contains Shanghai, Zhejiang province, Jiangsu province and Anhui province. JJJ is an important metropolitan area and includes Beijing, Tianjin two municipalities directly under the Central Government, and Hebei Province. CY is the center of China’s western development strategy, which includes most area of Sichuan and Chongqing. These three metropolitan areas are located in the northern hemisphere monsoon climate zone, with mild and dry winters, and hot and rainy summers. The three metropolitan areas include five National-level Central Cities of China (total eight cities honor the title) and play an extremely important role in regional leadership. They are located in China’s southeast (CSJ), north (JJJ) and southwest (CY) regions, their specific locations are shown in Fig. 10.

Due to economic development, the UBL in these three metropolitan areas has expanded significantly in the last fifteen years, and most was converted from cultivated land. After an overlay analysis of the LULC data, which was described in LUCC data section, we found that the UBL area grew 2346 km^2^ in CY from 2000 to 2015, with >90% converted from cropland (Table 3). The conversion rates in CSJ and JJJ were also more than 80%. From the point of view of change, IL was the main cultivated land transformed in CSJ, while DL accounted for the vast majority in JJJ. In CY, the converted proportions of the two types of arable land were similar. The proportional changes in different types of cultivated land is also related to their distribution^53^. The conversion of cultivated land to UBL resulted in the changes in surface characteristics and temperature. To clarify the temperature changes caused by cropland conversion to UBL in different geographical locations, we compared urban expansion warming effects in the three metropolitan areas from the quantitative and qualitative perspectives.

**Table 3:** Cultivated land and UBL transfer information in 2000–2015.

	CSJ	JJJ	CY
DL->UBL Area	1648 km^2^	1922 km^2^	863 km^2^
DL->UBL to DL->Other Area Percentage	82.03%	85.04%	48.19%
DL->UBL Grids	178	214	86
IL->UBL Area	6753 km^2^	245 km^2^	1266 km^2^
IL->UBL to IL->Other Area Percentage	83.84%	30.51%	77.01%
IL->UBL Grids	729	18	132
UBL Growth Area	9474 km^2^	2595 km^2^	2346 km^2^
Cropland->UBL to UBL Area Growth Percentage	88.67%	83.51%	90.75%
UBL Grids in 2000	2406	1360	278
UBL Grids in 2015	3373	1612	517

The area and ratio of conversion of dry and irrigated land to urban and built-up land are calculated by 1 km resolution remote sensing data, and the number of grids in the innermost domain of model simulation is counted by the remote sensing data resampled by the WRF Preprocessing System.

### Model configuration

The Weather Research and Forecasting model (WRF) is a next-generation mesoscale numerical weather prediction system, mainly designed by the National Center for Atmospheric Research (NCAR), the National Oceanic and Atmospheric Administration (represented by the National Centers for Environmental Prediction (NCEP) and many other institutions in the latter part of the 1990’s^54^. Because of the two dynamical solvers, Advanced Research WRF (WRF-ARW) and Non-hydrostatic Mesoscale Model (WRF-NMM), WRF is widely used in climate prediction and climate research^55^, which includes many relevant studies on warming effects caused by regional urbanization or temperature change caused by LULC in China^35,56^. The WRF (ARW) version 3.6.1, coupled with single-layer Urban Canopy Model (UCM) and Noah Land Surface Model (Noah-LSM), was used to simulate the urbanization processes from different cultivated land^57^. Different underlying surface scenarios can be substituted in the WRF preprocessing system (WPS) of the model so that different underlying surface parameters will drive the physicochemical module to simulate corresponding climatic results.

In this experimental design, the only variable is the fine-scale land use classification data of 2000 and 2015. The parameterization schemes of the key physical processes in all the simulation experiments are the same, and these schemes are summarized from experimental results and the literature. The WSM6 (WRF Single-Moment 6-class) is used for microphysics, the RRTM scheme and Dudhia scheme are set for longwave radiation and shortwave radiation. After four kinds of land surface schemes simulations for predictions of temperature and precipitation in China, the accuracy of the Noah land surface scheme was the highest. It also approved to be reliable in Jianjun, *et al*.^58^ study, so the Noah land surface model was the land-surface option we used. The detailed information of physical parameterization schemes are summarized in Table 4.

**Table 4:** WRF model physics and dynamic options.

Options	Parameterization Schemes
Microphysics	WSM6 (WRF Single-Moment 6-class)^71^
Longwave radiation	RRTM scheme^72^
Shortwave radiation	Dudhia scheme^73^
Surface-layer option	Revised MM5 Monin-Obukhov scheme^74^
Land-surface option	Noah land-surface model^75^
Urban surface option	Single-layer urban canopy model^75^
Boundary-layer option	YSU scheme^76^
Cumulus option	Kain-Fritsch scheme^77^

As far as possible to accurately simulate the meteorological conditions, each of the three metropolitan areas was simulated individually, rather than for the whole of China. The simulations were all configured with three levels of two way nested girds in the Lambert Project. The number of outermost grids of the three area simulations are 80 × 80, and grids in the innermost domain were 277 × 316 in CSJ, 214 × 277 in JJJ and 298 × 217 in CY. All contained the entire administrative divisions of each metropolitan area; detailed simulation area nested positions are shown in Fig. 10 and the number of DL2UBL and IL2UBL grids are shown in Table 3. All the simulations of three domains’ resolution are 27 km, 9 km and 3 km, and the vertical grid contains 26 levels from surface to 50 hPa.

**Figure 11:**
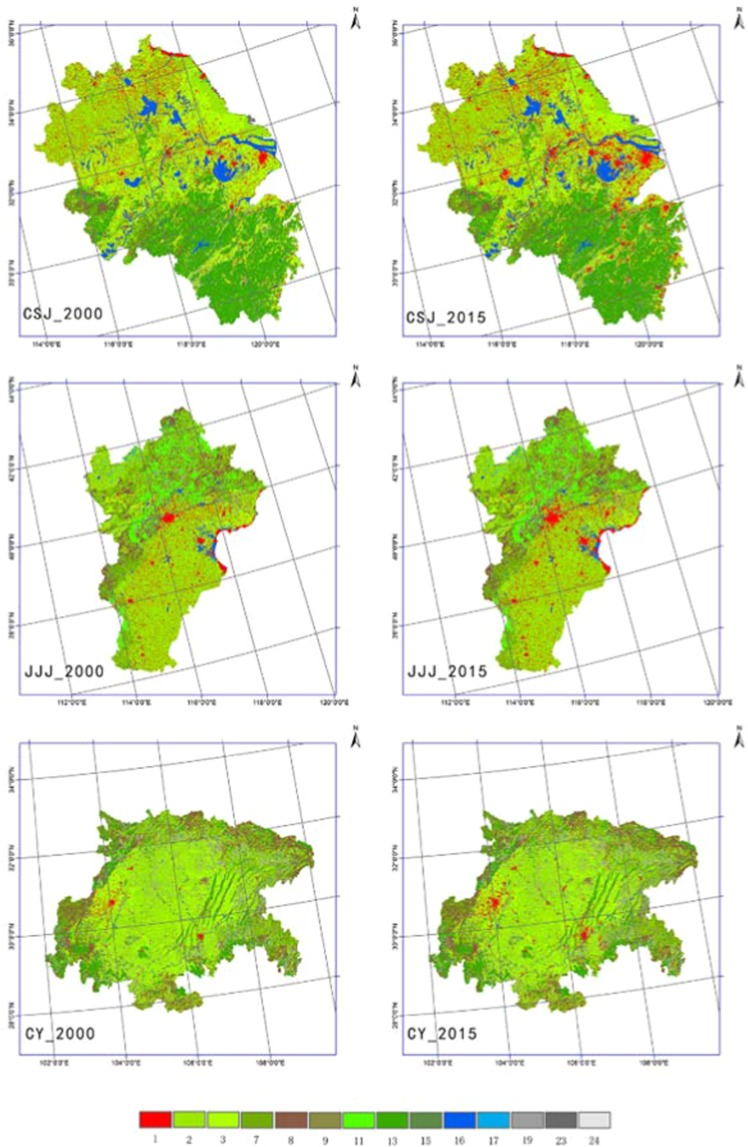
Land use and land cover of these three study areas in USGS classification. The top is the CSJ, the middle is JJJ, and the bottom is CY. The left three images are 2000 LULC, and the right refer to 2015. Number codes are: 1. Urban and built-up land, 2.Dryland cropland and pasture, 3.Irrigated cropland and pasture, 7. Grassland, 8. Shrubland, 9. Mixed grassland and shrubland, 11. Deciduous broadleaf forest, 13.Evergreen broadleaf, 15. Mixed forest, 16. Water bodies. 17. Herbaceous wetland, 19. Barren or sparsely vegetated, 23 Bare ground tundra, 24. Snow or ice.

**Figure 12:**
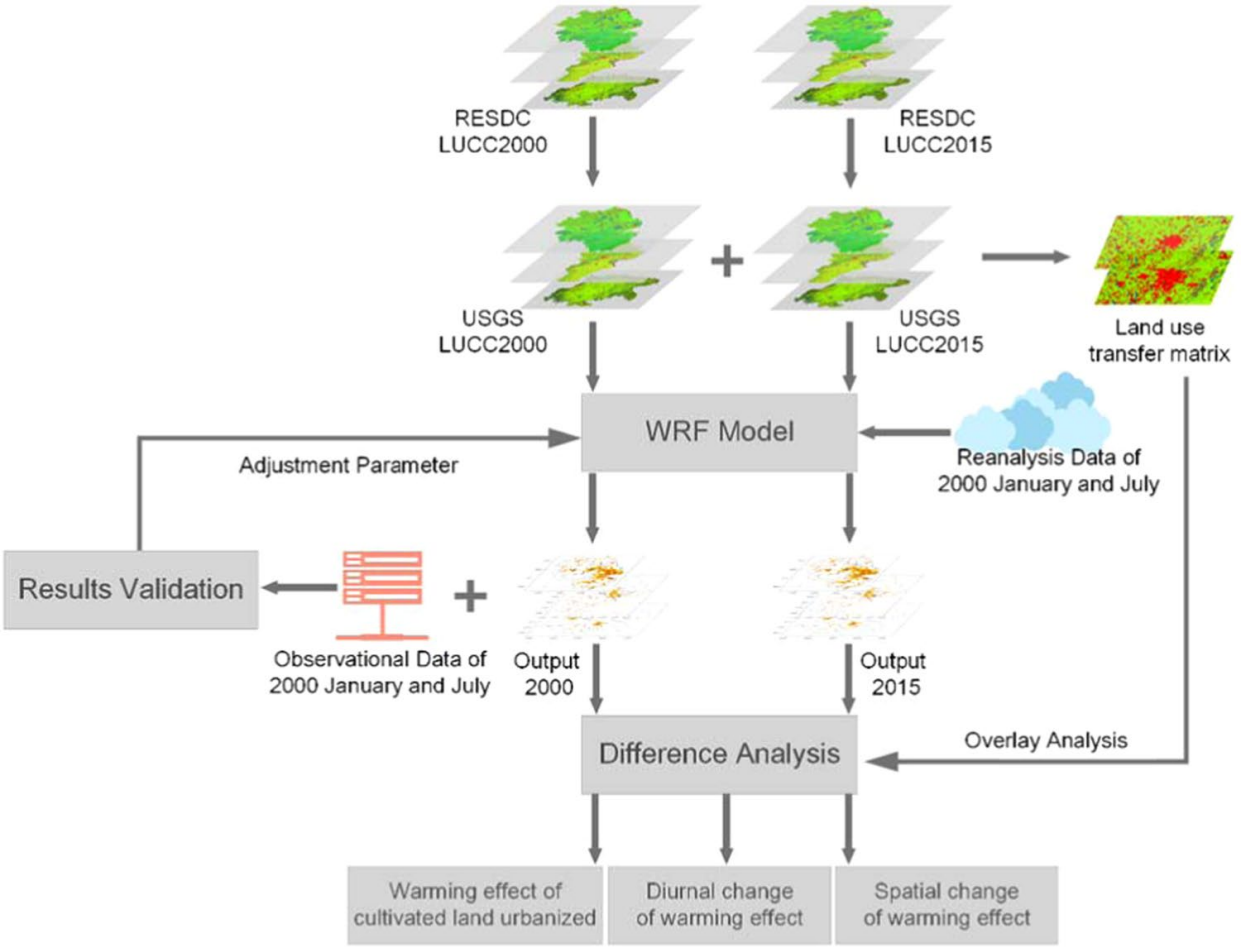
Schematic diagram of experiment flow in three metropolitan area regions.

Two LULC scenarios, conducted from RESDC (as described in LUCC data section), of 2000 and 2015 are configured in the model to represent before and after of the cultivated land change. To make the underlying land use data work in the model, and to optimize simulation time and error, the simulation length of each run was set one month from 2000 January 1st to 31st 2000 July 1st to 31st. We set these 2000 LULC scenarios as control experiment (CTL) and the 2015 scenario simulations as sensitive experiment (STV). The land-use maps used in CTL and STV are shown in Fig. 11. Each run started at 00:00 and first day of all cases are omitted from further analysis as they were viewed as spin-up time^59^. Our research objective was to determine dry land and paddy field temperature changes from converting to urban and construction land in different seasons. As winter and summer are the typical monsoon season of the region where the study area is located^17,60^, the experiment was conducted in January and July. The experimental diagrammatic sketch is shown in Fig. 12.

### Data resource and processing

#### LUCC data

As the only variable in the experiment, in order to accurately calculate the area of cultivated land converted into UBL, we use the fine classification LULC data from the Data Center for Resources and Environmental Sciences, belonging to Chinese Academy of Sciences (RESDC) (http://www.resdc.cn). The data, classified 6 first-level types and 25 secondary types, is based on the TM/ETM remote sensing image, and generated by artificial visual interpretation. This remote sensing monitoring database of land use plays an important role in the national land resource investigation, hydrology and ecological research in China^61–63^. The resolution of these LULC data sets are 1 km × 1 km, with the 94.3% of the first-level classified accuracy and 91.2% of the second-level’s^15^, which are satisfy the accuracy of simulation. However, the LUCC types are reclassified as USGS or MODIS classification types that WRF model can be recognized, so the standard of the type conversion we used is what conducted in our before experiment^19^. The LUCC classification of 26 RESDC classes were reasonably transformed into 24 USGS classes, and accurate results were obtained in simulation experiments. In the standard system, urban built-up land, rural residential land and other construction land of RESDC classification are conversed into urban and built-up land of USGS. The dry farmland of RESDC are conversed to dry cropland and pasture (DL) of USGS, and paddy land are categorized in irrigated cropland (IL). These three types of LUCC in the two sets of classification have similar standards, which ensures the clarity and accuracy of classification transformation. The Land use and land cover of these three study areas in USGS classification was shown in Fig. 11. The LULC transfer matrix is used to analyze the temporal and spatial variation of land use types, which carried out by raster calculator in ArcGIS10.2.

#### Elevation data

The LUCC data is not only the surface data that would affect the result of simulation in WRF, but also the elevation data. Both temperature and wind speed are sensitive to changes in surface elevation, because complex terrain has strong correlation with airflow and air turbulence, thus affecting the water-heat balance in the region^64^. There are three default digital elevation model (DEM) data in WRF, 30 arc sec, 2 arc min and 10 arc min, which represent about the resolution of 830 m, 3300 m, and 16600 m, respectively. Zhang, *et al*.^65^ compared these resolution with the Shuttle Radar Topography Mission (SRTM) data, 90 m (about 3 arc sec), in WRF simulation and found that the more precise DEM data, the smaller the error of temperature and wind speed of simulation results. So fine topographical data is important in WRF simulation, the DEM data of all domains in WRF are replaced by SRTM data in our experiments.

#### Reanalysis data

The reanalysis data is the data that uses the method of data assimilation to fuse all kinds of observation data and numerical forecast products as the initial condition of the later simulation^66^. It as initial data and boundary data drive WRF work in each domain. FNL reanalysis data of US National Centers for Environmental Prediction (NCEP) and the ERA series data of European Centre for Medium-Range Weather Forecasts (ECMWF) are widely used in WRF. The latest ERA-Interim data of ECMWF (http://apps.ecmwf.int/datasets/data/interim-full-daily/levtype=sfc/), used in this simulations with intervals of six hours (including 00:00, 06:00, 12:00 and 18:00 total four files a day), uses a relatively perfect numerical prediction model and assimilation system, also combines more remote sensing data and surface observations, so that it has been improved on the basis of the FNL data and original ERA data^66,67^. The January and July of 2000 ERA-Interim data are used to simulate with 2000 and 2015 LULC data, which control the variable, LUCC, as the only influence factor.

#### Observation data

The site observation data are used to verify the accuracy of WRF mode simulation results. This data sets, from the Meteorological Data Center of China Meteorological Administration (http://data.cma.cn/data/cdcdetail/dataCode/SURF_CLI_CHN_MUL_DAY.html), is based on the historical ground meteorological data construction project archived statistics, including the data of 824 basic stations in China from 1951 to 2010. There are 65 stations in CSJ, 27stations in JJJ and 30 stations in CY. The data is spatially transformed by using the geographic information system software arc GIS10.2 and overlapped with the WRF simulation results to validate.

### Surface heat content and effective temperature

The surface temperature is the metric to assess the warming effect, but it also requires assessments including latent heat of evaporation and humidity, a metric for surface heat content^24^. The heat content of surface near ground can be expressed as:

1 $${\rm{H}}={C}_{p}T+Lq$$

Where ${C}_{p}$ is the specific heat of air at constant pressure, 1005 J/(kg·°C) used in the calculation. T is the air temperature, regarded as the 2-m high air temperature (T2), $L$ is the latent heat of evaporation at 2-m, and $q$ is the specific humidity at a height of 2-m above ground^68^. As latent heat of evaporation is not a regular observed variable, in this research, Riedel’s method^69,70^ has been used to calculate the variable L. To compare it with the air temperature, the Eq. (1) is transformed to Eq. (2) ^24^:

2 $${T}_{{\rm{e}}}={H}_{e}/{C}_{p}$$

The value ${T}_{e}$, called effective temperature, represents the combination of humidity, latent heat of evaporation and temperature, and it can be expressed in units of Joules kg^−1^ (J/kg). In the analysis of DL2UBL and IL2UBL variations, it can be used to measure the warming effect, together with temperature, on heat content perspective.

## Data Availability

All data generated or analyzed during this study are included in the article.
